# Brain Functional Connectivity in *de novo* Parkinson's Disease Patients Based on Clinical EEG

**DOI:** 10.3389/fneur.2022.844745

**Published:** 2022-03-15

**Authors:** Matteo Conti, Roberta Bovenzi, Elena Garasto, Tommaso Schirinzi, Fabio Placidi, Nicola B. Mercuri, Rocco Cerroni, Mariangela Pierantozzi, Alessandro Stefani

**Affiliations:** ^1^Parkinson Centre, Department of Systems Medicine, Policlinico Tor Vergata, Rome, Italy; ^2^Neurology Unit, Department of Systems Medicine, Policlinico Tor Vergata, Rome, Italy

**Keywords:** Parkinson's disease, EEG, functional connectivity, graph theory, assortativity

## Abstract

In Parkinson's disease (PD), cortical–subcortical interplay plays a relevant role in affecting clinical performance. Functional MRI sequences described changes in functional connectivity at different stages of disease. Scarce are, instead, the investigations examining brain connectivity in patients with PD at early stages of disease. For this aim, here we analyzed the differences in functional connectivity between *de novo*, never treated, PD patients and healthy controls. The analyses were based upon custom-written scripts on the Matlab platform, combined with high-level functions of Fieldtrip, Brainstorm, and Brain Connectivity toolboxes. First, we proceeded to the spectral analysis of the EEG data in the five frequency bands (δ-θ-α-β-γ). Second, we calculated functional connectivity matrices based on both coherency (COH) and imaginary part of coherency (iCOH), in the δ-θ-α-β-γ frequency bands. Then, four network measures (density, transitivity, global efficiency, and assortativity) were computed in identified connectivity matrices. Finally, we compared the spectral density, functional connectivity matrices, and network measured between healthy controls and *de novo* PD patients through two-samples *T*-test. A total of 21 *de novo* PD patients and 20 healthy subjects were studied. No differences were observed in spectral analysis between the two groups, with the exception of the γ band where a significant increase in power density was found in PD patients. A reduced connectivity in the main EEG frequency bands (α-β frequency bands) was observed in PD patients compared to controls, while a hyperconnectivity was found in PD patients in γ band. Among the network measures, a reduced assortativity coefficient was found in *de novo* PD patients in α frequency band. Our results show the occurrence of early EEG functional connectivity alterations from the initial stages of PD. From this point of view, connectivity analysis may ease a better understanding of the complexity of PD physiopathology.

## Introduction

Parkinson's disease (PD) is a neurodegenerative disorder whose hallmark is the degeneration of dopaminergic neurons of pars compacta of substantia nigra, leading to the classic motor symptoms. However, the concept centered on an exclusive basal ganglia involvement does not completely explain the heterogeneous complexity of disease motor and non-motor symptoms spectrum. Therefore, new models, mainly based on prion-like spreading of misfolded α-synuclein from brainstem to subcortical and later cortical structures, were developed, suggesting a multisystem involvement in PD (1). Nevertheless, the clinical presentation of PD not necessarily follows the spatiotemporal pattern indicated by neuropathological findings. Underlying effects on brain networks by neuropathological changes might contribute to explain this phenomenon, even in the earliest stages of the disease.

In recent years, brain connectivity analysis was used successfully to better define the pathophysiology of dementias, mainly in Alzheimer's disease (2), with a predictive value (3). In this framework, cerebral connectivity analysis could be useful to improve the understanding also of the pathophysiological mechanisms of PD. So far, functional brain networks are usually examined by measuring the temporal correlations in functional MRI (fMRI) of blood oxygen level dependent (BOLD) signal between different brain regions (4). Electroencephalography (EEG) is a non-invasive and accessible method to evaluate the cortical electrical activity through scalp electrodes, routinely used in clinical practice for diagnoses of epilepsy or disturbances of consciousness. This technique may be used to estimate the functional interactions between brain areas, through different connectivity measures (5). Compared to fMRI, EEG has the advantage of direct measuring of electrical activity with high temporal resolution.

The aim of this study is to explore if dysfunctions in brain functional connectivity may feature PD since the early, off-therapy disease stages. For this purpose, we analyzed the EEG resting-state connectivity in *de novo* PD patients, compared to healthy controls, using a custom-written scripts on the Matlab platform, combined with high-level functions of Fieldtrip (6) and Brainstorm (7) toolboxes, common EEG analysis software, and Brain Connectivity toolbox, usually utilized for graph theory analysis (8). Preliminary data were presented at “7 Congresso Accademia LIMPE-DISMOV 2021”—December 15–17, 2021, Bologna, Italy.

## Materials and Methods

### Study Recruitment

We recruited patients diagnosed with idiopathic PD, according to the MDS clinical diagnostic criteria (9). Patients were required to meet the following criteria: (1) disease duration <24 months; (2) no history of taking therapy with dopaminergic drugs (iMAO, L-Dopa, Dopamine agonists, iCOMT, anticholinergic agents, amantadine); (3) no history of epilepsy or other conditions that could cause pathological alterations of EEG recording (i.e., brain tumors, stroke, infections, etc.); (4) no cognitive impairment, as defined by a Mini-Mental State Examination (MMSE) score above 25 (10); (5) morphological MRI without brain parenchymal lesions; and (6) no other neurological diseases except PD.

All included PD patients underwent the EEG recording session and, on the same day, they were clinically evaluated using the Unified Parkinson Disease Rating Scale motor section (UPDRS part III) (11).

As control group, we enrolled age-matched healthy subjects, without history of epilepsy or other conditions that could justify alterations of EEG, as described above for PD patients. Control cohort was composed of subjects under EEG scrutiny as part of diagnostic tests, which excluded epilepsy or other neurological diseases.

### EEG Acquisitions and Removal of Artifacts

EEG data were recorded for 10 min at a sampling rate of 128 Hz and band-pass filtered at 0.5–50 Hz using a 19-channel EEG system. Scalp electrodes were positioned according to 10–20 International System (12). Recording was performed during awake-resting state. Subjects were instructed to keep their eyes closed while staying awake. During the EEG acquisition, we monitored the level of vigilance of patients by visual inspection of EEG traces: in case of slowing of the EEG activity, sleepiness was avoided by giving instructions to the subjects once again.

After the acquisition, we selected a 100-s poor-artifact segment of each EEG recording. We used a cleaning algorithm for EEG data. First, as other authors have previously suggested, we applied detrending and re-reference to each channel (13). Then, we used independent component analysis (ICA), to remove EEG artifacts due to eye-blinks, muscle activity, cardiac signals, and line noise sources (14).

### Spectral Analysis

We proceeded to the spectral analysis of the EEG data. We applied the Welch's method, which consists in averaging consecutive Fourier transform, calculated using the Fast Fourier Transform (FFT) algorithm, of small windows of signal, with or without overlapping. In the present study, the EEG data were divided into segments of 1 s length, with overlap of 50%.

Then, we computed the power spectral density or periodogram for each subject in the five frequency bands (δ 0.5–4, θ 4–8, α 8–13, β 13–30, and γ 30–50 Hz).

### Connectivity Analysis

Coherency (COH) is a measure of the linear relationship of two EEG channels at a specific frequency. If *x*_*m*_(*f*) and *x*_*n*_(*f*) are the complex Fourier transforms of the time series *x*_*m*_(*t*) and *x*_*n*_(*t*) of the channel *m* and *n*, the cross-spectrum is defined as:


(1) $$G_{mn}\left(f\right)=\;{\langle x}_{m}(f)x_{n}^{*}(f)\rangle$$

Where ^*^ indicates the complex conjugation and 〈…〉 means the average value. Then, COH can be defined as the normalization of cross-spectrum:


(2) $$\begin{array}{l} COH_{mn}=\frac{|G_{mn}\left(f\right){|}^{2}}{G_{mm}\left(f\right)G_{nn}\left(f\right)}\; \end{array}$$

Where *G*_*mm*_(*f*) and *G*_*nn*_(*f*) are the power spectral density of *m* and *n*, respectively.

Coherency is widely used as a measure of EEG functional connectivity (15). In the present study, we considered COH and the imaginary part of coherency (iCOH), which is known to reduce the volume conduction artifacts, compared to COH (16). Functional connectivity matrices of all subjects were obtained by calculating the values of COH and iCOH between any pair of channels in the five fundamental frequency bands on segments of 1 s length, with overlap of 50%, according to the Welch's method.

### Network Measures

At this point, we analyzed the network properties. For this purpose, we computed the mean connectivity matrices of COH and iCOH of all subjects over the five frequency bands and calculated a cut-off value, so that 30% of all edges were considered significant. We thresholded the individual connectivity matrices by absolute weight, in order to generate weighted undirected networks.

We used four global metrics to measure network properties of all subjects [an extended description on measures of brain connectivity can be found on this paper (8)].

- Density: it represents the fraction of present edges to possible edges or “wiring cost” of the network.
- Transitivity: it is the ratio of triangles to triples in the network and represents a collective normalization of clustering coefficient (17). Transitivity is a classical measure of functional segregation in brain network, which can be define as the ability for specialized processing to occur within interconnected group of regions.
- Global efficiency: the functional integration in brain network represents the ability to rapidly combine information from different brain regions. Measures of integration are commonly based on the concept of a path: shorter paths imply stronger functional integration. Global efficiency can be define as the average shortest path length between all pairs of nodes in the network and it is commonly used as measure of functional integration (18).
- Assortativity: it is a correlation coefficient between the degrees of all nodes on two opposite ends of an edge (19). Networks with positive assortativity coefficient are likely to have a resilient core of mutually interconnected high-degree hubs, while networks with a negative assortativity coefficient are likely to have widely distributed and vulnerable high-degree hubs. Hence, assortativity represents an indirect measure of resilience, which reflects the network's vulnerability to insults.

**Figure 1:**
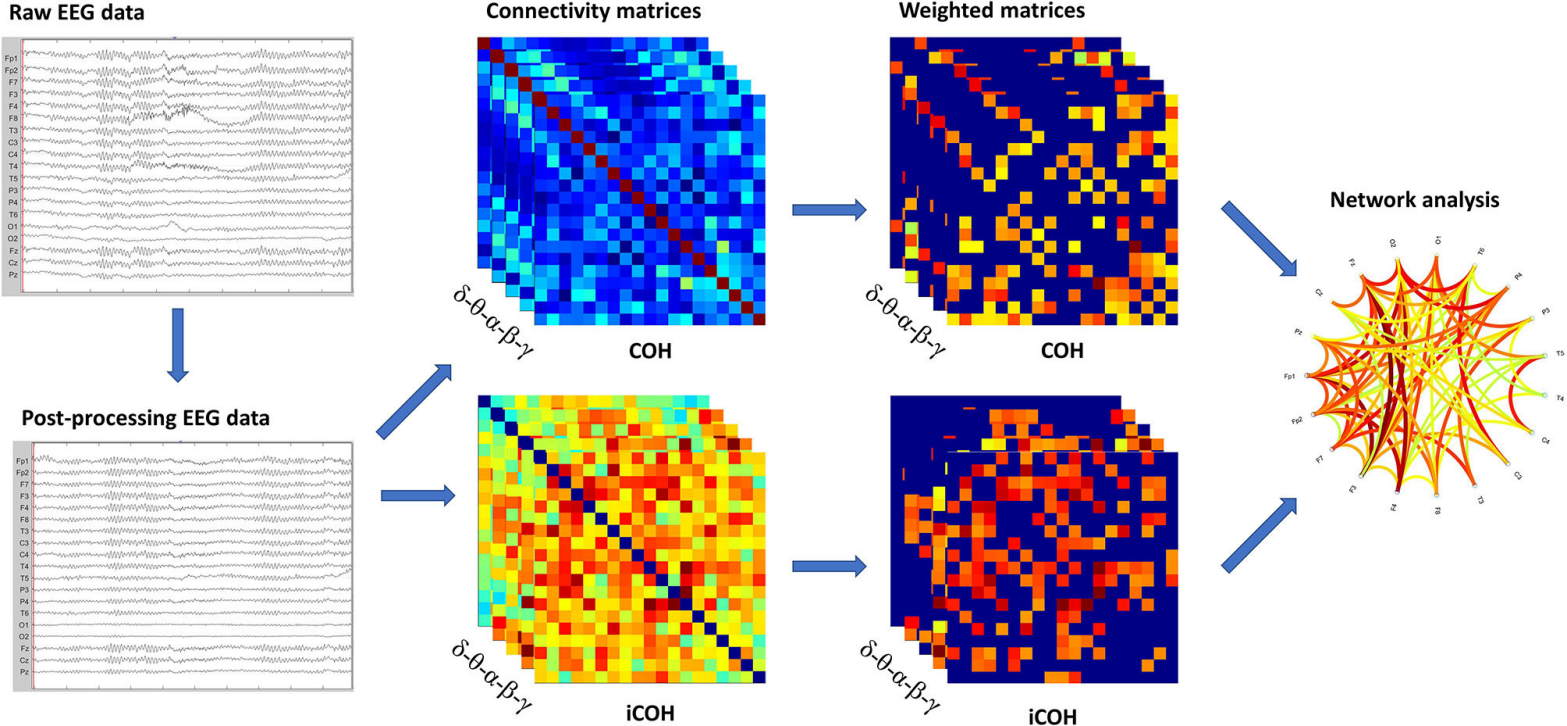
Schematic representation of the analysis. In sequence, we showed: raw EEG data filtered and cleaned using Independent Component Analysis (ICA); the functional connectivity matrices based on COH and iCOH for each frequency band; weighted undirected matrices based on coherency (COH) and imaginary part of coherency (iCOH) for each frequency band; graph representation of network.

A schematic representation of the different steps in the analysis is shown in Figure 1.

### Statistical Analysis

Since Kolmogorov–Smirnov test showed that variables followed a normal distribution, we performed parametric statistical tests. We analyzed the differences in the magnitude of power spectral density [i.e., sqrt (power)] for each EEG channel in the five frequency bands (δ-θ-α-β-γ) between *de novo* PD patients and healthy controls using two sample *T*-test. We analyzed the differences in the functional connectivity matrices based on COH and iCOH measures between *de novo* PD patients and healthy controls groups. We compared each cell of the matrices in the five frequency bands (δ-θ-α-β-γ) by two samples *T*-test. Finally, we compared global network measures of calculated connectomes (density, transitivity, global efficiency, and assortativity) between PD patients and controls using two samples *T*-test.

A preventive Levene's test was used for each *T*-test in order to verify if the populations examined had the same variance. Null-hypothesis was rejected at *p*-value < 0.05. The statistical analysis was performed using Matlab 2020a.

## Results

### Subjects

We used a population of 21 PD patients, who met the inclusion criteria of the study. They were consecutive patients, followed up to the Neurological Clinic of the University of “Tor Vergata,” Rome, between January 2020 and July 2021.

As control group, we enrolled 20 healthy subjects (see methods), who underwent the same EEG recordings of patients. The demographics and clinical characteristics of both groups are summarized in Table 1.

**Table 1:** Demographic and clinical characteristics of *de novo* Parkinson's Disease (PD) patients and healthy controls.

	***De novo* PD**	**Controls**
*N*	21	20
Sex (% male)	76.2	70.0
Age (years)	60.95 ± 10.47	60.45 ± 13.96
Disease duration (months)	8.48 ± 7.26	/
UPDRS III	14.4 ± 5.7	/

*Data are mean ± SD. UPDRS III, Unified Parkinson's Disease Rating Scale part III (motor symptoms)*.

### Comparison in Spectral Analysis

**Figure 2:**
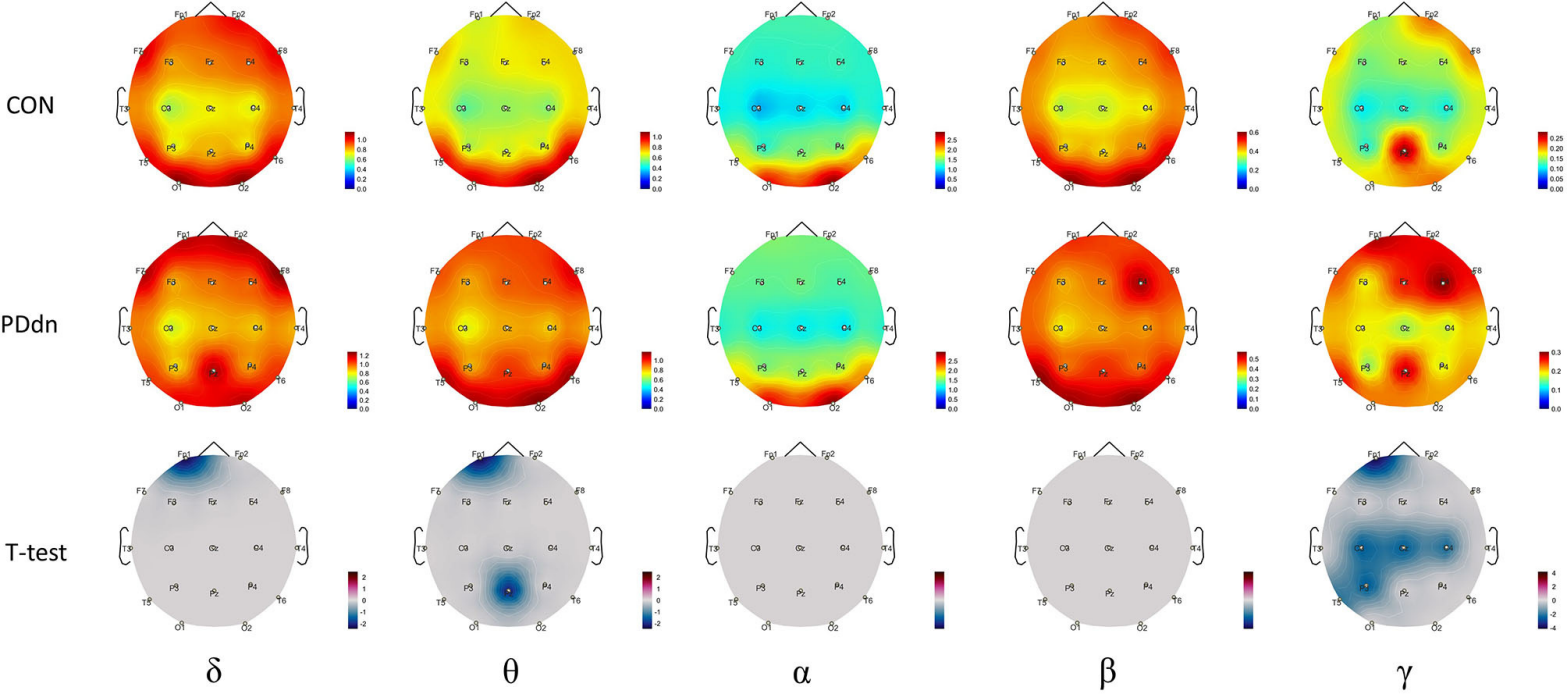
First and second rows show magnitude of power spectral density for each channel, respectively, in control and in PD de novo groups at δ-θ-α-β-γ frequency bands. Third row reports t-values between magnitudes of power spectral density of Controls and Parkinson's disease (PD) patients (CON-PDdn) for each channel at δ-θ-α-β-γ frequency bands. Only t-values with p-value < 0.05 are shown.

Comparisons in spectral analysis between PD patients and healthy controls are reported in detail in Figure 2. The spectral analysis documented an increased power density in PD patients compared to controls in δ-θ-γ frequency bands. In particular, we found a modest but statistically significant (*p*-value < 0.05) increase in power density in PD *de novo* patients in Fp1 channel in δ band and in Fp1 and Pz channels in θ band. A more extensive increase in power density was found in the γ band, affecting Fp1, C3, Cz, C4, and P3 channels. No statistically significant differences were found in α and β frequency bands.

### Comparison in Functional Connectivity

**Figure 3:**
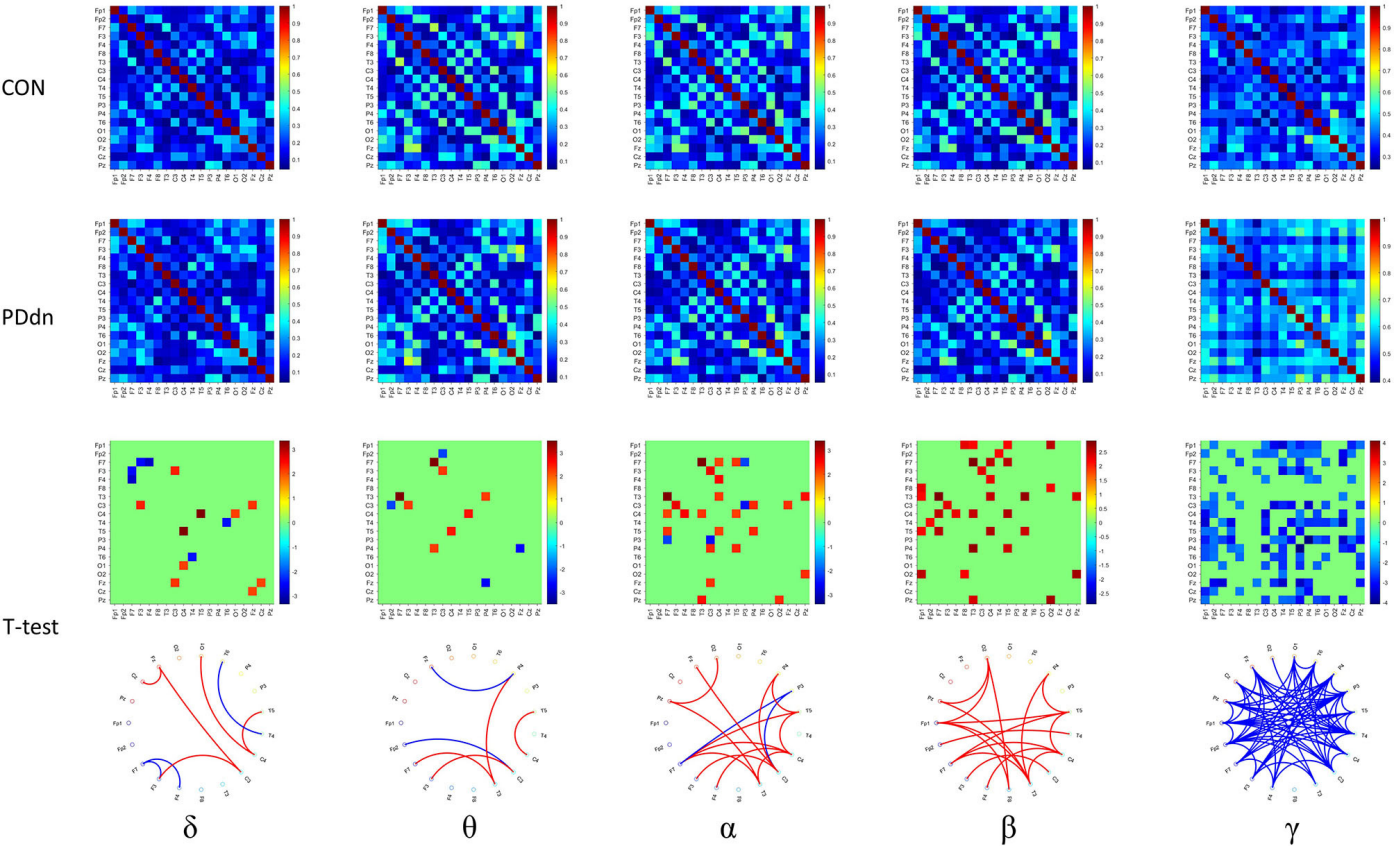
First and second rows show functional connectivity for each pair of channels based on COH, respectively, in healthy controls and in PD de novo patients at δ-θ-α-β-γ frequency bands. Third row shows t-values (CON-PDdn) and fourth row shows the graph representation of different functional connectivity for each pair of channels with a p-value < 0.05 at δ-θ-α-β-γ frequency bands between controls and PD patients (red indicates higher connectivity in healthy controls, while blue in PD patients).

First, we analyzed the functional connectivity based on COH (Figure 3). In the δ-θ frequency bands, there is no clear predominance of functional connectivity in controls or in PD patients. In α and β, we observed a significant reduced functional connectivity or hypoconnectivity in PD patients compared to healthy controls. Instead, we found a widely increased functional connectivity in PD patients compared to controls in the γ frequency band.

**Figure 4:**
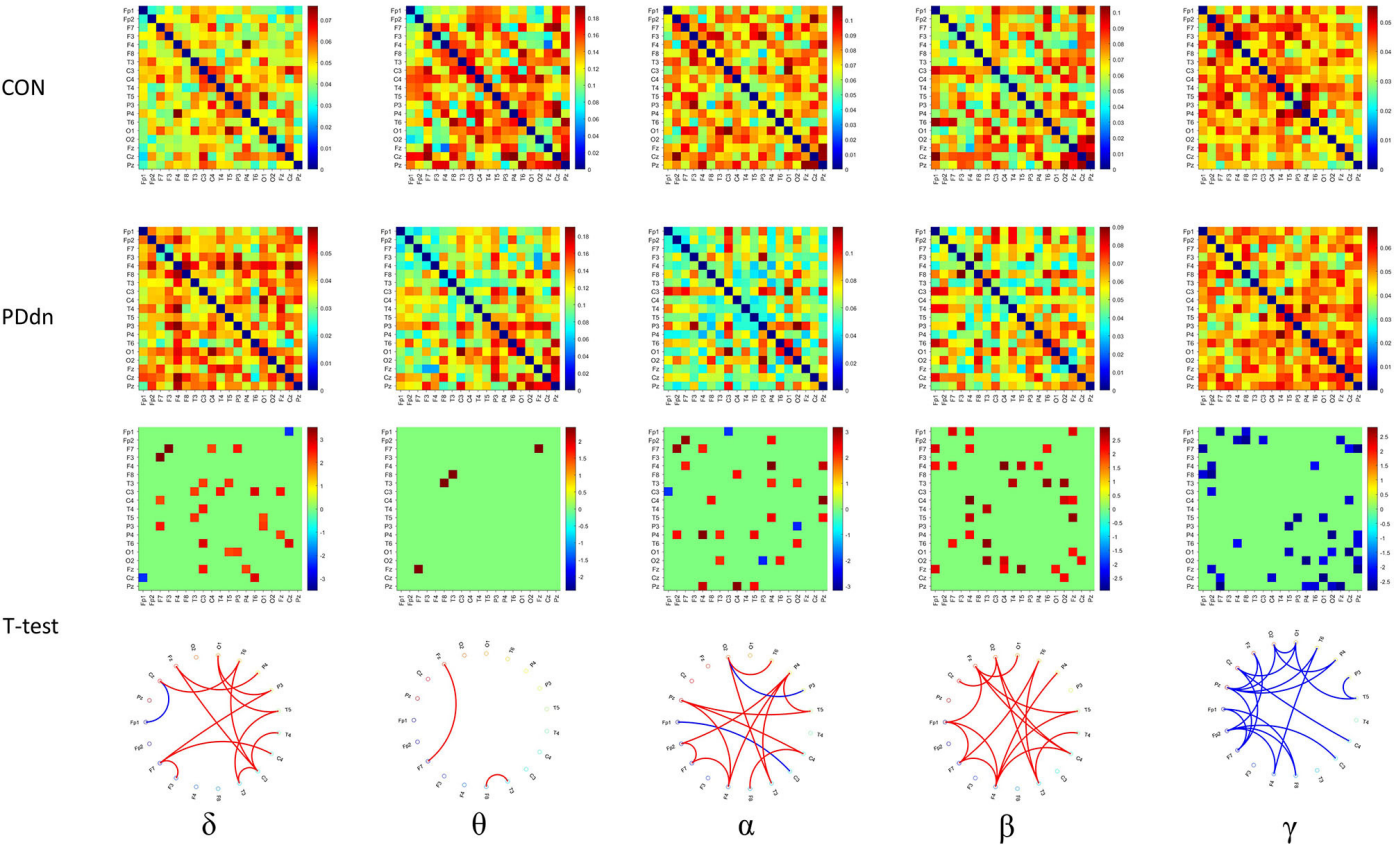
First and second rows show functional connectivity for each pair of channels based on iCOH, respectively, in healthy controls and in PD de novo patients at δ-θ-α-β-γ frequency bands. Third row shows t-values (CON-PDdn) and fourth row shows the graph representation of different functional connectivity for each pair of channels with a *p*-value < 0.05 at δ-θ-α-β-γ frequency bands between controls and PD patients (red indicates higher connectivity in healthy controls, while blue in PD patients).

Similar results were also observed in functional connectivity based on iCOH (Figure 4). Compared to the connectivity based on COH, a clear increase in connectivity can be found in controls in δ band, while no substantial differences were observed in θ band. A clear hyperconnectivity is confirmed in healthy controls compared to PD patients also in connectivity based on iCOH in α and β, as well as an increased connectivity was observed in PD patients in γ frequency band, even if it appeared statistically significant in a smaller number of pairs of channels compared to connectivity based on COH.

### Differences in Network Measures

**Figure 5:**
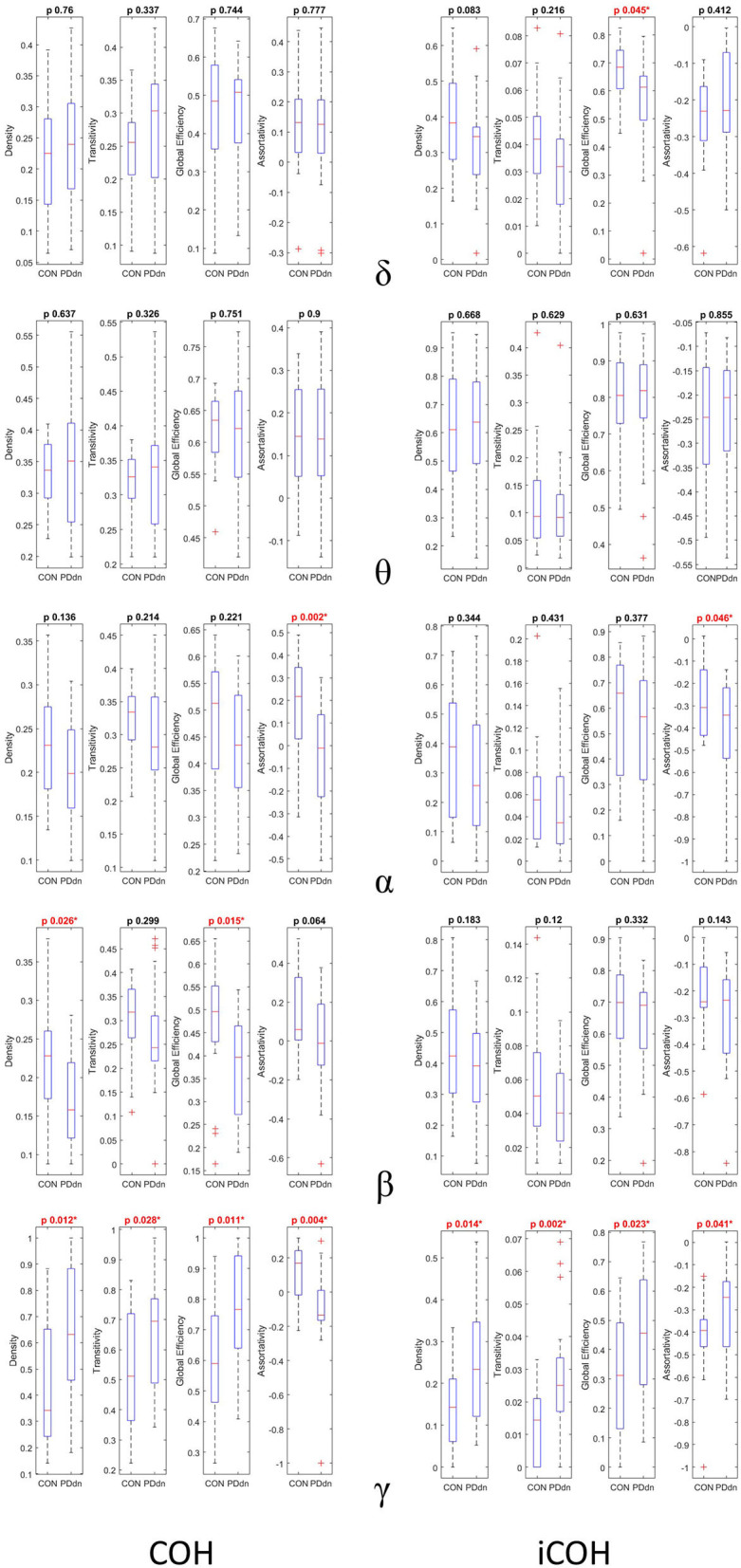
First and second columns show box plots and p-value of two samples T-test (CON-PDdn) of four network measures (density–transitivity–global efficiency–assortativity) between healthy controls and PD patients at δ-θ-α-β-γ frequency bands of functional connectivity matrices based on, respectively, COH and iCOH. Red color indicates a p-value < 0.05).

We compared four network measures (density, transitivity, global efficiency, and assortativity) between controls and PD patients of functional connectivity matrices based on COH and iCOH. Comparisons can be found in detail in Figure 5.

No significant differences were observed between control and PD groups in δ and θ bands in COH- and iCOH-based connectomes. In α band, a higher assortativity coefficient was found in controls than in PD patients both in COH (*p*-value 0.002) and in iCOH (*p*-value 0.046) based connectomes. In COH-based connectivity increased density (*p*-value 0.028) and global efficiency (*p*-value 0.015) in controls were found in β band, while the assortativity coefficient resulted higher in controls than in patients with *p*-value at the limits of significance (*p*-value 0.064). No significant differences were found in β band in iCOH-based connectomes.

An inverse result was observed in γ frequencies. In iCOH based-connectivity, all four network measures resulted higher in PD patients compared to controls (density-transitivity-global efficiency-assortativity; *p*-value 0.014, 0.002, 0.023, 0.041). In COH-based connectivity increased density, transitivity and global efficiency were found in PD patients (*p*-value 0.012, 0.028, 0.011), while assortativity coefficient resulted higher in control group (*p*-value 0.004).

## Discussion

### Dysfunction in α-β Bands Functional Connectivity

The aim of this study was to assess the functional connectivity integrity in *de novo* PD patients compared to healthy controls. We found that PD patients in the early stages of disease have a reduced connectivity in the main EEG frequency bands (α-β), contrary to the spectral analysis, which did not reveal any significant differences in α-β bands. These data seem to suggest that changes in functional connectivity may precede alterations in spectral analysis in PD patients. Indeed, a previous study showed a reduced power spectral density in α-β bands in advanced PD patients (20). We can, therefore, hypothesize that spectral analysis modifications do not characterize the initial stages of disease, contrary to the reduced functional connectivity.

Previous studies based on fMRI have reported functional connectivity alterations in PD patients, mainly in sensorimotor network (SMN) and in default-mode network (DMN). SMN is a large-scale brain network, involved in performing and coordinating motor tasks (21), and its alteration has been largely reported in PD (22–25). The DMN is a network consistently active during resting-state or task-negative conditions, and it is involved in a large number of functions, such as thinking about themselves or others, remembering past events or perception of time (26), and dysfunction in DMN has been observed in cognitively impaired patients with PD (27–29).

Compared to the previous fMRI studies, our study is based on EEG recordings, non-invasive and accessible method to evaluate cortical electrical activity. Our data are, therefore, interesting considering the pathophysiology of PD, since we analyzed PD patients in the first stages of disease in which the synucleinopathy should not have reached the cortical areas, according to the Braak theory (1). In first hypothesis, the dysfunction we observed in EEG functional connectivity may be related to downstream remote effects, i.e., through striatal-thalamocortical circuits (30), rather than local cortical involvement. This is in line with previous studies demonstrating that synucleinopathy can alter the resting-state functional networks as consequence of deficits in other brain regions (22). On the other hand, recent hypothesis of top-down cortical pathogenesis of PD was proposed (31), as opposed to Braak's theory of bottom-up progression. Therefore, from this point of view, the alteration of EEG functional connectivity documented in our study could be evidence of cortical involvement since the early stages of PD.

### Assortativity Coefficient in *de novo* PD Networks

Among the network measures, interesting results derive from the analysis of the assortativity coefficient, which was found to be reduced in *de novo* PD patients in α frequency band both in COH- and iCOH-based connectivity. Assortativity coefficient represents the correlation between degrees of all nodes on two opposite sides of an edge, and is an indirect measure of the network resilience (19).

In assortative networks, nodes with higher degrees tend to be connected together, so the disturbed connections of one node can be compensated by other high-degree nodes. Indeed, in assortative networks, high-degree and low-degree vertices tend to link to other high-degree and low-degree vertices, respectively. When the assortativity coefficient decreases, this order is altered, and some nodes start establishing new connections with vertices with less similar degrees to their own degrees. The lower assortativity coefficient, we observed in PD patients, might indicate that some cortical regions start establishing or increasing connections with other regions to compensate an initial deficiency. However, an analysis of the sources is necessary to corroborate this hypothesis.

### Compensatory Hyper-Connectivity in γ Frequencies

A further result of our research is that PD patients showed a hyper-connectivity in γ frequency band, when compared to other frequency bands. Previous studies have demonstrated that γ oscillations of cortical neurons are generated by interactions between pyramidal cells and inhibitory interneurons, and reflect synchronization phenomena (32, 33). The γ oscillations have been associated with movements executions and with planning of movements (33–37). The γ band has also been related to sensory and cognitive processing, long-term memory, and language tasks (38, 39).

In our opinion, the observed γ hyper connectivity may be interpreted as compensatory mechanism performed by cortical neurons still not affected by synucleinopathy. In other words, it should be presented in the early stages of PD and be lost as the disease progresses, accompanied with a worsening of the motor symptoms. Actually, a previous study based on extracellular recordings of subthalamic nucleus neurons of advanced PD patients during surgery for deep brain stimulation, has demonstrated that percentage of units oscillating at γ frequency was negatively correlated with the bradykinesia scores (40). Moreover, it was observed a positive correlation between γ band power and the improvement of symptoms with Levodopa administration (41), which could indicate a better response to dopaminergic therapy in PD patients due to the integrity of the cortical γ connectivity. Furthermore, a recent intermittent theta burst stimulation (iTBS) study emphasized the role of cortical γ oscillations in the pathophysiology of the abnormal long-term potentiation (LTP)-like plasticity in PD patients (42), indicating a positive correlation of cortical γ oscillations with synaptic plasticity.

Finally, a previous experimental study that analyzed cortical and pallidal γ frequency in hemiparkinsonian rats with unilateral 6-hydroxydopamine (6-OHDA) lesion, showed that γ band increased only in animals manifesting levodopa-induced dyskinesias (LIDs) (43). Future studies are needed to demonstrate the potential predictive role of early γ hyperconnectivity in the subsequent development of LIDs.

### Limitations of the Study

The study is limited by relatively small number of PD patients and healthy controls. This limitation is partly due to the strict inclusion criteria, restricted to *de novo* drug-naive PD patients, and with exclusion of cognitive impairment history. However, a larger number of patients may ensure a better representation of the statistical sample.

As mentioned earlier, the study is limited by the low resolution of the EEG, as the study is based on standard recordings, which can only allow sensor-based connectivity analysis, and it is not possible to carry out an adequate reconstruction of the brain sources due to the number of channels (44). However, it has the advantage of allowing a functional connectivity analysis through an easily accessible and safe tool in the Neurology departments.

Finally, the present study is not longitudinal, yet. It may be useful to follow-up patients over time to observe further changes in EEG brain functional connectivity.

### Conclusions

Functional connectivity analysis may ease a better understanding of the complexity of PD physiopathology, from the earliest stages of disease. We found a reduced functional connectivity in *de novo* PD patients in α and β frequency bands both in COH and in iCOH-based connectivity analysis, suggesting their dysfunction in a disease stage, in which LP should not have reached the brain cortical areas. We also found a paradoxical hyperconnectivity in the γ band, which we interpreted as an initial compensation mechanism by brain areas spared by extensive LP. Future studies are needed to unleash the potential of a widespread functional connectivity analysis on PD patients.

## Data Availability Statement

The raw data supporting the conclusions of this article will be made available by the authors, without undue reservation.

## Ethics Statement

The studies involving human participants were reviewed and approved by Ethical Committee of Policlinico Tor Vergara. The patients/participants provided their written informed consent to participate in this study.

## Author Contributions

MC: conceptualization, data curation, formal analysis, methodology, software, and writing – original draft. RB: formal analysis and methodology. EG: data curation. TS: visualization. FP: validation. NM: visualization. RC: resources. MP: writing – review & editing. AS: supervision and writing – review & editing. All authors contributed to the article and approved the submitted version.

## Funding

This manuscript received contribution from BRIC 2019 (INAIL) to AS and RF-2018-12365509 to AS and NM.

## Conflict of Interest

The authors declare that the research was conducted in the absence of any commercial or financial relationships that could be construed as a potential conflict of interest.

## Publisher's Note

All claims expressed in this article are solely those of the authors and do not necessarily represent those of their affiliated organizations, or those of the publisher, the editors and the reviewers. Any product that may be evaluated in this article, or claim that may be made by its manufacturer, is not guaranteed or endorsed by the publisher.
